# Acute Appendicitis Together with Chylous Ascites: Is It a Coincidence?

**DOI:** 10.1155/2010/206860

**Published:** 2010-05-31

**Authors:** Sami Akbulut, Davut Yilmaz, Sule Bakir, Erdal Cucuk, Mahmut Tas

**Affiliations:** ^1^Department of Surgery, Diyarbakir Education and Research Hospital, Op. Dr. Seref Inaloz Caddesi, 21400 Diyarbakir, Turkey; ^2^Department of Pathology, Diyarbakir Education and Research Hospital, Op. Dr. Seref Inaloz Caddesi, 21400 Diyarbakir, Turkey; ^3^Department of Emergency Medicine, Diyarbakir Education and Research Hospital, Op. Dr. Seref Inaloz Caddesi, 21400 Diyarbakir, Turkey

## Abstract

Acute chylous ascites is a rarely seen clinical picture, therefore, examination findings are often confused with acute appendicitis. To the best of our knowledge, there is no publication to date showing the occurrence of them together. This study presents the treatment plan for a 25-year-old male patient with both acute chylous ascites and appendicitis. Surgical findings were retrocaecal appendicitis, evident lymphangiectasia in the proximal segment of jejunum, and approximately 3 lt of chylous fluid. An appendectomy was performed and drainage was applied. Low-fat total parenteral nutrition (TPN) and octreotide treatment were administered for 7 days postoperatively. We also present a general review of some studies on chylous ascites, which have been published in the English language medical literature since 1910.

## 1. Introduction

Chylous ascites is a milky or creamy fluid, rich in triglycerides, which accumulates in the peritoneal cavity [1–6]. Extravasation of chylous fluid into the peritoneal cavity is rarely seen, thus, the clinical signs may be interpreted as an acute or a chronic condition depending on the rate of the fluid flow. Malignancy, trauma, cirrhosis, and tuberculosis account for 95% of the causes for chylous ascites [7]. Because of the wide range of etiologies, there is still no consensus among surgeons on the approach to chylous peritonitis.

This study aimed to give the clinical presentation of a 25-year-old male patient, who was admitted for surgery with signs and symptoms of both acute appendicitis and acute chylous peritonitis. We also give an overview of the studies on chylous ascites published in the English language medical literature and until now, no causal link has been detected between chylous ascites and appendicitis.

## 2. Case Report

**Figure 1:**
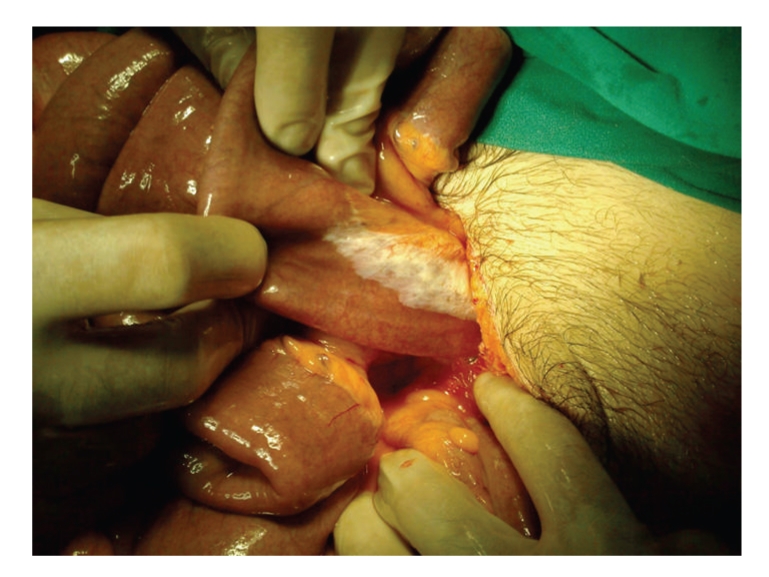
Milky white lymphangietasic area of the jejunal segment immediately distal to the Treitz ligament, seen from the left and the right.

**Figure 2:**
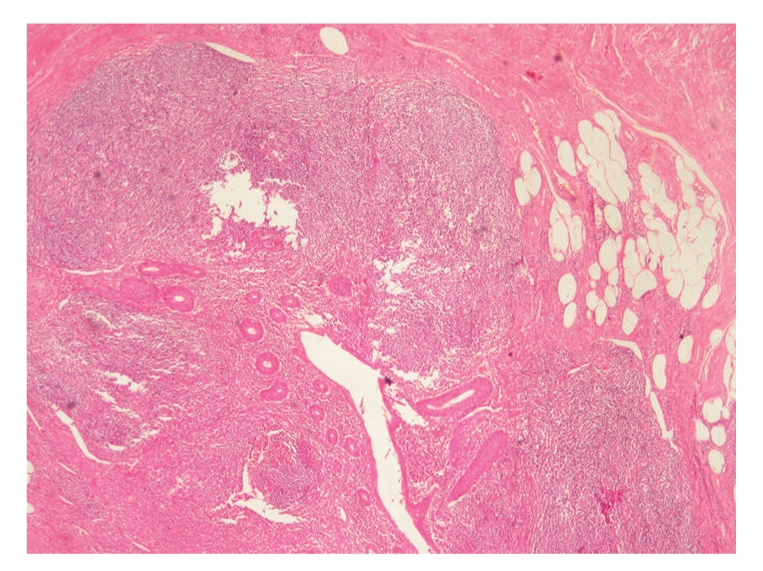
A light mononuclear inflammation was observed to have infiltrated the cells of the muscle and serous layer in the submucosal appendix tissue (x40 H&E).

A 25-year-old male presented to the emergency department with abdominal pain, bloating, nausea, and vomiting. According to his history, the abdominal bloating and pain had started 3 days before and the patient had been prescribed painkillers, proton pump inhibitors (PPIs), and antiflatulence medication. In the morning of the day he came to our clinic, the patient had complaints of nausea and vomiting, and widespread abdominal pain developed. During the day, the pain had become more localized in the lower right quadrant and he presented to our clinic around 09.00 pm. The patient reported absence of bowel movements for 2 days. The patient history showed no alcohol or tobacco use, no chronic illness, and no trauma or surgical intervention. Physical examination revealed a temperature of 38°C, moderate dehydration, abdominal distension, and definite rebound in the lower right quadrant. On auscultation the intestinal bowel sounds were slightly reduced and digital rectal examination showed an empty ampulla. Laboratory results were WBC 12.400 K/UL (4.3–10.3), haemoglobin 13.8 gr/dL (13.5 17.2), BUN 55 mg/dL (10–45), and creatinine 0.98 mg/dL (0.6–1.2). AST, ALT, ALP, GGT, amylase, lipase, HDL, LDL, triglyceride, and albumin values were within normal limits. The abdominal ultrasonography revealed minimal fluid between intestinal segments in the lower right quadrant and in the pelvic area. The abdominal radiograph showed several small areas of air and fluid. Due to these findings, the patient was admitted for surgery with an initial diagnosis of perforated appendix. The abdomen was opened with a McBurney incision. When the peritoneum was opened, milky peritoneal fluid was observed. Then the retrocaecal appendix was freed and due to its inflammation, appendectomy was performed. However, after the aspiration of 2.5–3 lt of fluid, a perforation of an abdominal organ was presumed, and midline incision was made into the abdomen. The milky fluid was seen in all quadrants of the abdomen. After aspiration of the fluid, on the wall of the proximal jejunal segment 10 cm distal to the Treitz ligament, an expanded lymphatic network with a lymphangiectasic structure was seen (Figure 1). Two drains were placed in the abdomen and the operation was finished. Total parenteral nutrition (TPN) and Octreotide (Sandostatin-Novartis) 4 × 0.1 mg sc were administered for 7 days postoperatively. The drains were removed on postoperative day 5 and 7. On postoperative day 10, the patient was discharged without any complications. Bacteriological examination of the fluid showed no growth. The histopathological examination of the appendix showed acute appendicitis (Figure 2). At the end of postoperative 1 month, endoscopy and abdominal computed tomography (CT) were performed and neither of these examinations showed any pathological findings. 

## 3. Discussion

Chylous ascites is a rare form of ascites resulting from the collection of lymphatic fluid in the abdominal cavity and is seen in 1 out of 20,000 patients presenting to hospital [2–4]. Chylous ascites can be defined as acute or chronic depending on the rate of the fluid flow into the peritoneal cavity.

**Acute Chylous Ascites**

It occurs when there is no underlying disease and findings of peritoneal irritation develop due to the rapid collection of fluid in the peritoneal cavity. Patients suffer from loss of appetite, nausea, vomiting, and pronounced abdominal pain related to the peritoneal irritation. Biochemical and microbiological analyses are necessary in this situation [3, 6].

**Chronic Chylous Ascites**

It generally appears clinically without pain and with increasingly distended abdomen. Symptoms as loss of appetite, weakness, and abdominal pain are often seen but not specific. As the fluid collects slowly, the abdominal cavity adapts, and complaints are tolerated by the patients [1, 6, 8].

When we look at the literature, acute ascites is seen less frequently in comparison with the chronic form [9]. In a published compilation of 140 cases of chylous ascites by Vasko et al., 14% of adolescents and 21% of children had acute chylous ascites [10]. Our study observed an acute development phase.

The etiology of chylous ascites can be congenital or acquired. *Congenital Reasons*: for example, chyle cysts, congenital lymphangiectasia, lymphatic hypoplasia, and idiopathic reasons; the majority of cases of chylous ascites in childhood are in this group. *Acquired Reasons:* for example, malignant diseases, after abdominal or thoracic surgery, abdominal tuberculosis, pancreatitis, filariasis, inflammatory diseases such as retractile mesenteritis, blunt or penetrating trauma, volvulus, obstructions caused by herniation and bridges, cirrhosis, hemodynamic malfunctioning due to cardiac, and renal insufficiency; they are more often seen in adults. The reasons are well summarized in the table of the study entitled “Causes of chylous ascites” by Smith et al. [1]. Three different mechanisms may take place in the development of chylous ascites.

1. Obstruction of the lymph flow caused by external pressure (mass) leading to leakage from dilated subserosal lymphatics into the peritoneal cavity.
2. Exudation of lymph through the walls of dilated retroperitoneal vessels lacking valves, which leak fluid through a fistula into the peritoneal cavity as in congenital lymphangiectasia.
3. Traumatic thoracic duct obstruction causing direct leakage of chyle through a lymphoperitoneal fistula [4, 6, 11].

A comparison based on geographical characteristics as an etiological factor showed that in Europe and North America, two-thirds of all patients had malignant diseases and cirrhosis, whereas in South America and Asia, tuberculosis and infectious diseases such as filariasis were more frequently seen [4, 7, 11].

In a study by Lerrick et al. [6], 30 cases of chylous peritonitis reported between 1910 and 1953 were analysed. Most patients were operated on for trauma, acute appendicitis, obstruction, herniation and clinical volvulus. Some of the notable cases of that study are a 4-month pregnant woman, followed without surgery and treated by paracentesis postpartum; a one-month-old infant who underwent surgery for volvulus; a male patient who died as he had been late in seeking medical care.

According to Thompson et al. [12], a total of 57 cases of acute chylous peritonitis were published in the English language medical literature between the first case reported by Renner in 1910 and 1981. 

Holcomb et al. [5] noted a total of 62 published cases of acute chylous peritonitis between 1910 and 1988. Of these, 8 cases were children, 5 boys, and 3 girls, aged between 1 month and 15 years. According to Holcomb, 35 were idiopathic, 13 obstructive, 8 traumatic, and 6 developed due to cyst rupture. When the studies by Holcomb and Lerrick are compared, it can be seen that there was a significant increase in cases of spontaneous chylous peritonitis after 1953.

According to Hardy et al. [13], there were a total of 63 cases of acute chylous peritonitis published in the English language literature between 1910 and 1992.

Liu et al. reviewed in a study 10 cases of chylous ascites (6 chronic, 4 acute) developing secondary to pancreatitis between 1953 and 2001 [9]. Later than that study, we found reports of 4 cases of chylous ascites secondary to pancreatitis between 2001 and 2009 [1, 7, 11, 14].

 Acute chylous ascites with no trauma history or other specific agent is referred to as spontaneous chylous ascites. Most cases of spontaneous chylous ascites in the literature occur in young adults and they are generally admitted for surgery with a diagnosis of acute appendicitis [1, 6]. In cases of extravasation chyle in the retroperitoneal area, the fluid is most frequently collected in the right paracolic gutter and therefore, in this type of patients, the pain is more evident in the lower right quadrant. In our case, most of the fluid had been collected in the lower right quadrant. In the English language medical literature, there are five studies in which acute appendicitis had been clinically mimicked, but in all of them the appendix was normal and no explanation could be found for the clinical manifestation of chylous ascites [1, 3, 13, 15, 16]. In the light of this, to the best of our knowledge, we are presenting the first case of actual appendicitis together with chylous ascites. The characteristics of these studies are summarized in Table 1.

The most important stage of the diagnosis is preoperative suspicion, then the radiological and biochemical results can be of benefit. Abdominal fluid can be seen on USG performed in the emergency department, but the clinician may interpret this as a perforated appendix. Blood biochemical analysis may not show excessive changes, so the most important diagnostic tool is paracentesis, which can be of considerable value in making the diagnosis of chylous ascites if the fluid is milky or creamy, triglycerides >2.24 mmol/L and glucose <5.5 mmol/L [1, 3, 4]. In our case, the triglyceride concentration in the ascites fluid was 12.6 mmol/L, which was much higher than the triglyceride concentration in the blood. A limitation of our study was that diagnostic paracentesis was not performed in order to differentiate the bleeding, perforation, regular ascites.

Theoretically, CT, lymphangiography, and lymphoscintigraphy can also be used. However, according to the studies in the literature, most patients were evaluated under emergency conditions and the decision about surgery was made. It is not possible to carry out all these diagnostic tests under emergency conditions. A significant proportion of cases of chylous ascites has developed in cases of cirrhosis of the liver and intra-abdominal malignancies, therefore, these tests can be useful for observation during the chronic period [4].

The choice of medical approach to the disease depends on the etiology. Under emergency conditions, a simple paracentesis can relieve pressure in the abdomen, prevent renal function failure, make the patient more comfortable, and give the clinician the opportunity to analyse the fluid for diagnosis. Patients with acute abdominal findings should be investigated immediately. Laparotomy is the first choice to confirm the diagnosis, to explain any underlying causes and for the necessary treatment to be applied [1, 3, 4]. The first step for all cases of chylous ascites, except for surgical acute abdomen, should be conservative medical treatment. The basic aim of treatment in this group of patients is to improve the nutritional deficit, to relieve the intestines and to decrease the rate of chylous fluid production. In the postoperative period, cases with long-term chylous drainage together with chronically persistent ascites, the use of middle-chain fatty acids or low-fat TPN can be of benefit [1, 3–5, 7, 11]. TPN can be used to achieve complete bowel rest and might allow resolution of the chylous ascites. Multiple case reports describe the use of octreotide in the management of chylous ascites. Somatostatin receptors have been described in the lymphatic vessels of the intestine, which may explain the efficacy of octreotide in the management of chylous ascites due to its contribution to decrease the lymph flow through these vessels [1, 3, 7, 17]. Our patient had an excellent response, with complete resolution of ascites, within the first week of adding TPN to the octreotide injection.

In conclusion, although chylous ascites and appendicitis are rarely seen together, when there is a suspicion of ascites findings on abdominal examination, a simple paracentesis and immediate USG can direct the appropriate treatment plan.

## Figures and Tables

**Table 1:** A summary of five cases of chylous peritonitis presenting as acute appendicitis reported in English Literature.

	References	Year	Age	Sex	Leucocyte Count	Appendix	Other Operative Finding	Surgical Procedure	Peritoneal Fluid Trigliseride Level
1	Smith et al.	2009	38	M	Normal	Not inflamed	Acute pancreatitis	Only exploration	26 mmol/L
2	Sah et al.	2009	19	M	Normal	Not inflamed	mixed germ cell tumor in the mesenteric LAP	Laparoscopic appendectomy	
3	Fang et al.	2006	22	M	11.600	Not inflamed	lymphatic leakage from the thoracic duct	Suture ligation	5.48 mmol/L
4	Fazili et al.	1999	25	M	20.000	Not inflamed	Normal	Prophylactic appendectomy	8.60 mmol/L
5	Hardy et al.	1992	26	M	14.300	Not inflamed	Lymphatic leakage from the small bowel mesentrey	Prophylactic appendectomy	15 mmol/L
6	Present report	2009	25	M	12.400	Severe inflamation	Proximal jejunal lymphangiectasia	Appendectomy+ drainage	12.6 mmol/L

## Abbreviations

TPN: Total parenteral nutrition
CT: Computed tomography
USG: Ultrasonography
PPI: Proton pump inhibitor.
